# The First Genomic Characterization of the Chikungunya Virus in Saudi Arabia

**DOI:** 10.1007/s44197-023-00098-0

**Published:** 2023-04-08

**Authors:** Hassan I. Alguridi, Faisal Alzahrani, Hisham N. Altayb, Safar Almalki, Eitezaz Zaki, Saeed Algarni, Abdullah Assiri, Ziad A. Memish

**Affiliations:** 1 Molecular Biology Department, Jeddah Regional Laboratory, Ministry of Health, P.O. Box: 17040, Jeddah, 21484 Saudi Arabia; 2 Department of Biochemistry, Faculty of Sciences, King Abdulaziz University, Jeddah, Saudi Arabia; 3 Centre for Artificial Intelligence in Precision Medicine, King Abdulaziz University, Jeddah, Saudi Arabia; 4 King Fahd Medical Research Center, Embryonic Stem Cells Unit, King Abdulaziz University, Jeddah, Saudi Arabia; 5 Laboratories and Blood Banks Administration, Ministry of Health, Jeddah, Saudi Arabia; 6 Public Health Authority, Jeddah, Saudi Arabia; 7 Deputy Ministry for Public Health, Ministry of Health, Riyadh, Saudi Arabia; 8 Research and Innovation Center, King Saud Medical City, Ministry of Health, Jeddah, Saudi Arabia; 9 College of Medicine, Alfaisal University, Riyadh, Saudi Arabia; 10 Hubert Department of Global Health, Rollins School of Public Health, Emory University, Atlanta, GA USA

**Keywords:** Chikungunya, ECSA, E1: K211E, E2: V264A

## Abstract

**Background:**

Chikungunya is an arboviral infection caused by the Chikungunya virus (CHIKV) transmitted to humans by mosquitoes of *Aedes* spp. CHIKV has been confined to African countries and South-East Asia up to 2004, but since then, the pathogen has become more global, and its high morbidity rate has become more visible. Saudi Arabia is not an endemic region of CHIKV, and the virus’s origin is not yet fully understood. This study aimed to characterize the genome of CHIKV from samples detected in Jeddah in 2018.

**Method:**

Twenty-two sets of primers were designed to amplify near-full length genome of CHIKV. RT-PCR was conducted from clinical samples. Two samples were used for studying near complete genome sequence while the remaining samples were used to study the E1 gene. Different bioinformatics tools were utilized.

**Results:**

Phylogenetic analysis showed that the CHIKV strains clustered with strains isolated from Kenya during 2017–2018 and belonged to ECSA genotype. E1: L136F, K211E and I317V mutations were identified in our strains. Also, E2: M74I, A76T, and V264A mutations were documented. Additionally, the capsid N79S substitution was also detected.

**Conclusion:**

The genome of CHIKV was analyzed for the first time in Saudi Arabia to better understand the origin of the CHIKV and its genetic diversity, which showed high similarity with IE—a subclade of CHIKV strains detected in Mombasa (Kenya) indicating its possible origin.

**Supplementary Information:**

The online version contains supplementary material available at 10.1007/s44197-023-00098-0.

## Introduction

Chikungunya is a Makonde term meaning “that which bends up”, which describes the infected patient’s contorted posture caused by severe joint pain. Chikungunya fever, which is classified as an arboviral infection, is caused by the Chikungunya virus (CHIKV). It is commonly transmitted by mosquitoes [1], mainly by females of the species *Aedes aegypti* [2]*.* Transmission occurs primarily during the day but can also happen at night [3]. CHIKV was reported for the first time in 1952–1953 in Tanzania [4]. Chikungunya has been confined to African countries and South-East Asia up to 2004 and is now considered an emerging infection with worldwide distribution. The virus spread from African countries into numerous countries (more than forty) throughout Africa, Asia and Europe; there have also been over one million CHIKV cases in the Americas since 2014 [5].

Throughout the past 50 years, several CHIKV re-emergences were reported in Asia and Africa with sporadic intervals from 2–20 between them. During the reported outbreaks, accurate figures of infected individuals could only be estimated due to the absence of serological surveillance. CHIKV emerged in Kenya and then spread to Comoros in 2004, and 5000 cases were documented. Between 2005 and 2006, outbreaks of CHIKV occurred on certain islands in the Indian Ocean, including La Réunion (France). During this epidemic, an estimation of 237 deaths were reported among 300,000 cases of CHIKV infection. A genetic investigation regarding CHIKV showed the linkage between the outbreaks on Réunion and Kenya. Moreover, the epidemic spread to India, where it was estimated that more than 1.5 million incidents occurred. Subsequently, CHIKV was identified in the United States and Europe and was supposedly attributed to infected travelers returning from infected areas [1].

CHIKV belongs to the genus alphavirus of the *Togaviridae*, which includes Ross River virus (RRV), Semliki Forest virus (SFV) and Sindbis virus (SINV). Alphaviruses are classified as spherical enveloped (icosahedral capsid). CHIKV possesses a single-stranded positive-sense RNA genome. This genome is made up of about 12,000 nucleotides, which encode five structural proteins and four non-structural proteins (nsP1–4) essential for virus replication. The structural proteins are glycoproteins E1, E2, E3, 6 K and capsid (C) protein [5].

CHIKV causes several signs and symptoms, unlike Dengue or Zika viruses, as it is commonly linked to a high rate of symptomatic infection. Symptoms of infection include headache, high fever, fatigue, myalgia, rash, myocarditis, recurrent polyarthralgia [6], muscle pain and abdominal pain [2]. Importantly, in vulnerable people such as neonates and the elderly, further severe symptoms may develop such as ocular diseases, pneumonia, fulminant hepatitis, neurological disorders and hemorrhage [2]. Furthermore, maternal infection during gestation may lead to fetal and neonatal illness [7].

Phylogenetic studies have shown that CHIKV can be classified into three geographical lineages: Asian, East/Central/South African (ECSA) and West African [6]. Since 2005, the ECSA lineage, which selected the E1 A226V mutation, was reported to be spreading worldwide. This mutation allows the virus to adapt and replicate in *Aedes albopictus* [8]. African genotypes show a broad range of diversity, while Asian genotypes exhibit higher levels of conservation [9]. Since CHIKV re-emerged in India and other regions in the Indian Ocean, many studies have been conducted to characterize it [6].

In the Kingdom of Saudi Arabia (KSA), CHIKV was reported for the first time in 2011 in Jeddah (western region) as a case report of a 55-year-old female. The polymerase chain reaction (PCR) diagnosis was followed by partial sequencing of the CHIKV E1 gene to detect the A226V mutation. The analysis showed that Jeddah CHIKV 2011 strain was a wild type [10]. Moreover, research was conducted to study the prevalence of CHIKV in the southern region of KSA (Asir and Jazan regions) using reverse transcription-polymerase chain reaction (RT-PCR) and enzyme-linked immunoassay (ELISA). The study included forty serum samples collected from patients based on rheumatological profiles and hematological parameters between December 2019 and February 2020. Analysis of RT-PCR and ELISA results of the forty samples showed that only one case was considered positive with anti-CHIKV IgG antibodies, and CHIKV RNA was not detected in any of the collected samples [11].

KSA is not an endemic region of CHIKV, and the virus’s origin is not yet fully understood. To date, no study has been conducted on the CHIKV whole genome in KSA according to our search on PubMed and Google Scholar for articles regarding this perspective (until July 2022). Therefore, investigation of the CHIKV genome in KSA may help to better understand the origin of the virus and its emergence; furthermore, it might enrich the knowledge of the genetic nature of the virus. This retrospective study aims to characterize the genome of CHIKV from CHIKV-positive samples detected in Jeddah in 2018. The objectives include determining this emerging virus's circulating genotype and mutation signature.

## Methods

The study was conducted in collaboration with the Faculty of Sciences (Biochemistry Department) at King Abdulaziz University, Jeddah, Regional Laboratory (Ministry of Health) and King Fahd Medical Research Centre.

### Sample Collection

A total of 21 serum samples (CHIKV RNA extracts) were provided retrospectively by Jeddah Regional Laboratory, a specialized lab for diagnosing viral diseases. All provided samples were tested utilizing CHIKV real-time PCR. These samples were detected previously in Jeddah in 2018 and stored at − 80 °C. Based on the guidelines of the used diagnostics kit LightMix Chikungunya Virus RT-qPCR (TIB MOLBIOL) in combination with FastStart DNA Master HybProbe master mix kit (Roche diagnostics–Germany), results of CT value 36 and below were considered positive.

### RNA Extraction and Detection of CHIKV by Real-Time PCR

CHIKV real-time PCR was utilized to test and confirm positive CHIKV from the provided samples. The viral RNA was extracted by an automated instrument: MagNA Pure 96 system (Roche diagnostics-Germany). A total of 50 μL RNA elution was extracted from 200 μL human serum samples. One-step real-time RT-PCR was performed using RealStar Chikungunya RT-PCR kit 2.0 (Altona Diagnostics–GmbH, Hamburg). The test was conducted according to the kit manufacturer's instructions. All 21 provided samples were tested.

### Complementary DNA (cDNA) Synthesis

cDNA was synthesized from the RNA extracted directly from each provided clinical sample (serum). The extracted CHIKV RNA was reverse transcribed into cDNA. Transcriptor First Strand cDNA Synthesis Kit (Roche diagnostics–Germany) was utilized to create cDNA according to the manufacturer's guidelines. Random Hexamer Primer was recruited and a volume of 5 μL (118 ng/ μL) extracted CHIKV RNA was used.

### Designing of PCR Primers

Twenty-two sets of primers (Table S1 and Figure S1) were designed to cover (and overlap) the near-full length genome of CHIKV. The primers were designed based on several (longest) reference sequences (MF773563.1, LN898112, MG921596.1, MG208125.1, KT192707.1, KR046234.1 and KY415985.1) obtained from the National Center for Biotechnology Information (NCBI). NCBI Primer-BLAST, Oligonucleotide Properties Calculator and Sequence Manipulation Suite were used for primer design and to check the quality of the generated primers. A further set of (nested) primers (Table S2 and Fig. S1) was designed to be utilized for a second-round PCR to amplify a fragment of the E1 gene of CHIKV from samples that showed low viral load (late-cycle threshold) as during this study, clinical samples (serum) were used directly without any viral culture. This set of primers can amplify an inner fragment of the CHIKV genome located inside the area of the genome that can be amplified by the forward primer of set number 20 and the reverse primer of set number 21 that amplifies the E1 gene. All primers were manufactured by Macrogen company (South Korea).

### CHIKV Genome Amplification by PCR

Among all provided samples, two samples showing the highest DNA quantity namely HF-793 and HF-777 were used for studying the near-full length genome sequence. Hence, all twenty-two sets of primers were used to amplify twenty-two regions of the virus, which represent the near-full length genome. The remaining 19 samples were consumed to study the E1 gene, which is commonly studied in the phylogenetic evaluation of CHIKV [6] and CHIKV genotype classification [12]. To perform the PCR reaction, FastStart DNA Master HybProbe master mix kit (Roche diagnostics–Germany) was used according to the manufacturer’s guidelines. An amount of 5 μL of each cDNA was utilized in a total reaction volume of 20 μL. All PCR yields were visualized by Agarose gel (1%) electrophoresis.

### CHIKV Genome Sequencing and Analysis

Genome sequencing was conducted using Sanger methodology, targeting both directions of the twenty-two amplicons that were produced from each sample (HF-793 and HF-777) via the PCR reactions. The DNA sequences were analyzed using FinchTV program (http/ /www.geospiza/products/finchtv.stml) that allows to view DNA sequence files and assess their quality from the chromatograms. The quality values of the nucleotides were in the Q30-Q60 range. The generated sequences were aligned and assembled using Snap-Gene software (Chicago, IL, USA);, the BLAST was used then for sequence identification. Partial sequencing of the E1 gene was conducted by sequencing bi-directionally each amplicon that was produced from the 5 samples (HF-116, HF-145, HF-653, HF-937 and HF-1442). Similarly, each pair of bi-directional sequences were assembled and assessed to confirm CHIKV by NCBI BLAST. Bacterial and Viral Bioinformatics Resource Center (BV-BRC) was used for genome annotation. Multiple sequence alignment and phylogenetic tree construction were achieved using Molecular Evolution Genetics Analysis (MEGA 11) Software [13]. A phylogenetic tree was created based on the near-full length genome sequences of CHIKV from this study (HF-777 and HF-793) in addition to 34 CHIKV sequences of all genotypes obtained from NCBI, using the neighbor-joining option in MEGA11. Bootstrap phylogeny test was used (1000 replicates). An additional phylogenetic tree was generated based on the E1 gene, including all 7 Jeddah strains and 25 others published CHIKV isolates from different genotypes. The E1 gene amino acid variations were estimated by the alignment of amino acids of the strains in this study and in the most similar Kenyan isolate (MT380161.1) with known isolate (HQ456251.1) as a reference of ECSA, using DNASTAR software. Additionally, an estimation of E2, E3 and C amino acid variations was conducted on samples that produced near-full length genome (HF-777 and HF-793).

## Results

### Detection of CHIKV by Real-Time PCR

CHIKV RNA was detected in all 21 provided samples. Testing these samples with real-time PCR showed different cycle thresholds (CT). The lowest CT value was 17, which might indicate a high virus titer in the sample, while the highest CT was 36 (Table S3).

### CHIKV Genome Amplification by PCR

PCR amplification of two positive samples with the highest DNA quantity showed all expected bands, which indicated the successful amplification of CHIKV. Each band (Figure S2) represents a part of the CHIKV genome. Therefore, all 22 PCR products (1–22 amplicons) represent near-full length genome of the virus. Furthermore, PCR amplification of the E1 gene from the 19 remaining samples showed a band of interest (1267 bp) only in 4 samples (HF-116, HF-653, HF-937 and HF-1442). A faint band (1267 bp) was detected in sample number HF-145, which was re-amplified by nested PCR to produce a 530 bp target. Out of the processed samples, we were able to sequence 7 samples only. The remaining samples were unsuitable for sequencing due to low viral loads (Table S3).

### CHIKV Genome Sequencing and Analysis

Regarding the whole genome, a total of 44 sequences were generated from sequencing the 22 PCR amplicons bi-directionally of sample HF-793 by Sanger technology. Evaluation of each of these sequences through NCBI BLAST revealed that all forty-four sequences were fragments of the CHIKV. Assembly of the forty-four sequences (by Snap-Gene software) produced the near-full sequence of the identified virus, which was 11,492 nucleotides. Further assessment of this genome sequence using NCBI BLAST confirmed the Chikungunya virus. Annotation of the obtained complete sequence (11,492 nucleotides) revealed a total of 9 genes (representing 5 structural and 4 non-structural proteins). These genes are E1 envelope glycoprotein, 6 K membrane protein, E2 envelope glycoprotein, E3 protein, capsid protein, RNA-directed RNA polymerase nsp4, non-structural protein nsp3, protease nsp2, and putative mRNA-capping enzyme nsp1, C-terminal (Fig. S3). Similarly, analysis of the genome sequences that were assembled and generated from the identified virus of sample number HF-777 showed 11,097 nucleotides. Partial sequencing of the E1 gene from 4 samples, HF-116, HF-653, HF-937 and HF-1442, produced 1161, 1162, 1159 and 1159 nucleotides, respectively. Moreover, partial sequencing of the E1 gene from sample HF-145 using second-round PCR had a final sequence of 519 nucleotides. Identification of the strains in this study showed high similarity (Table S4). All sequences acquired from the present study have been submitted to Genbank with accession numbers OQ230626-OQ230631, ON734069 and ON734070.

**Fig. 1:**
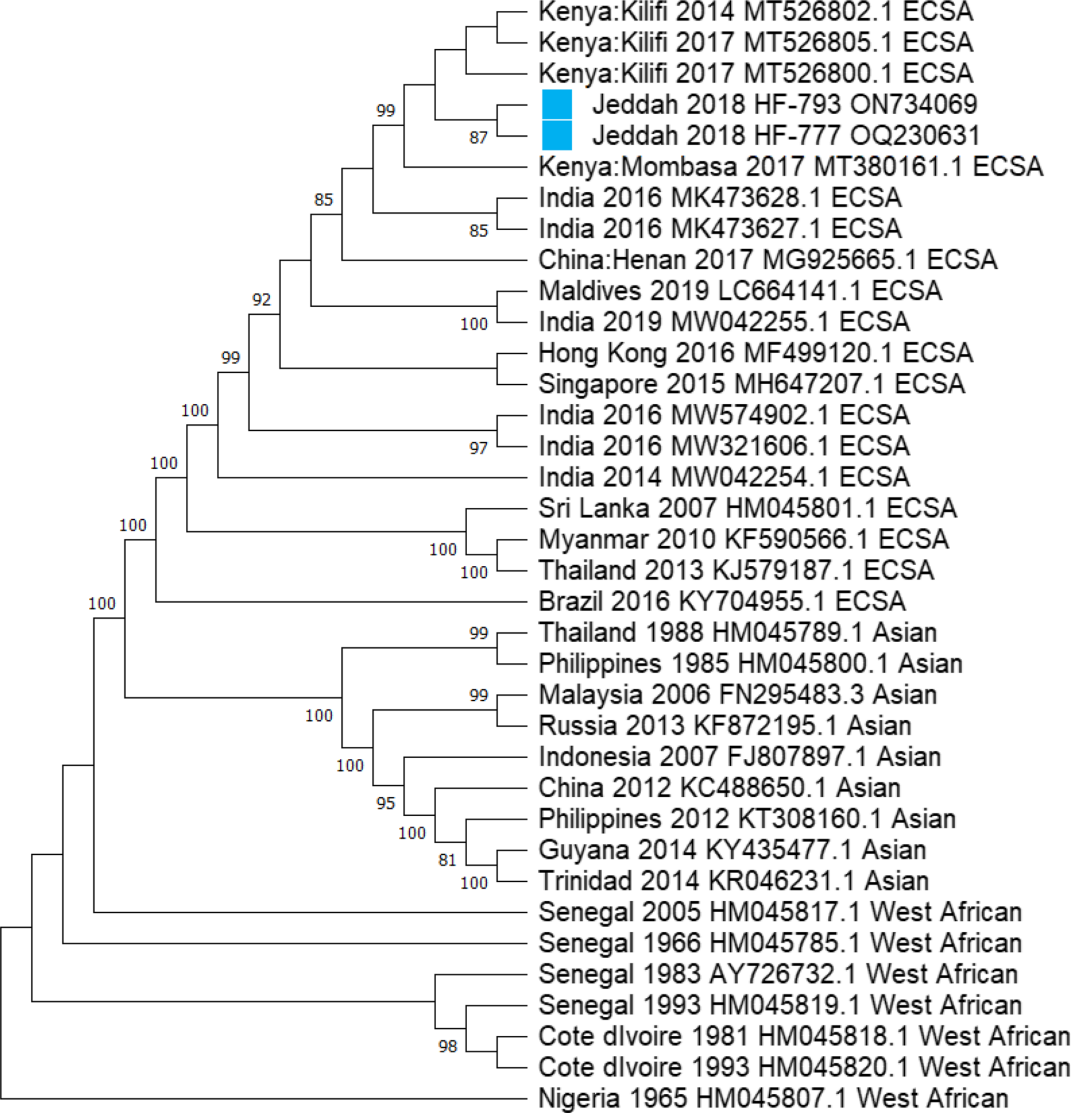
Phylogenetic tree of CHIKV (near-full length genome) strains from Jeddah during 2018. The phylogenetic tree was constructed using the neighbor-joining option in MEGA11and Bootstrap phylogeny test was used (1000 replicates). Jeddah strains, which were specified with a light blue square, clustered with strains isolated from Kenya mostly during 2017. Percent bootstrap support is indicated by the values at each node (the values < 80 are omitted)

**Fig. 2:**
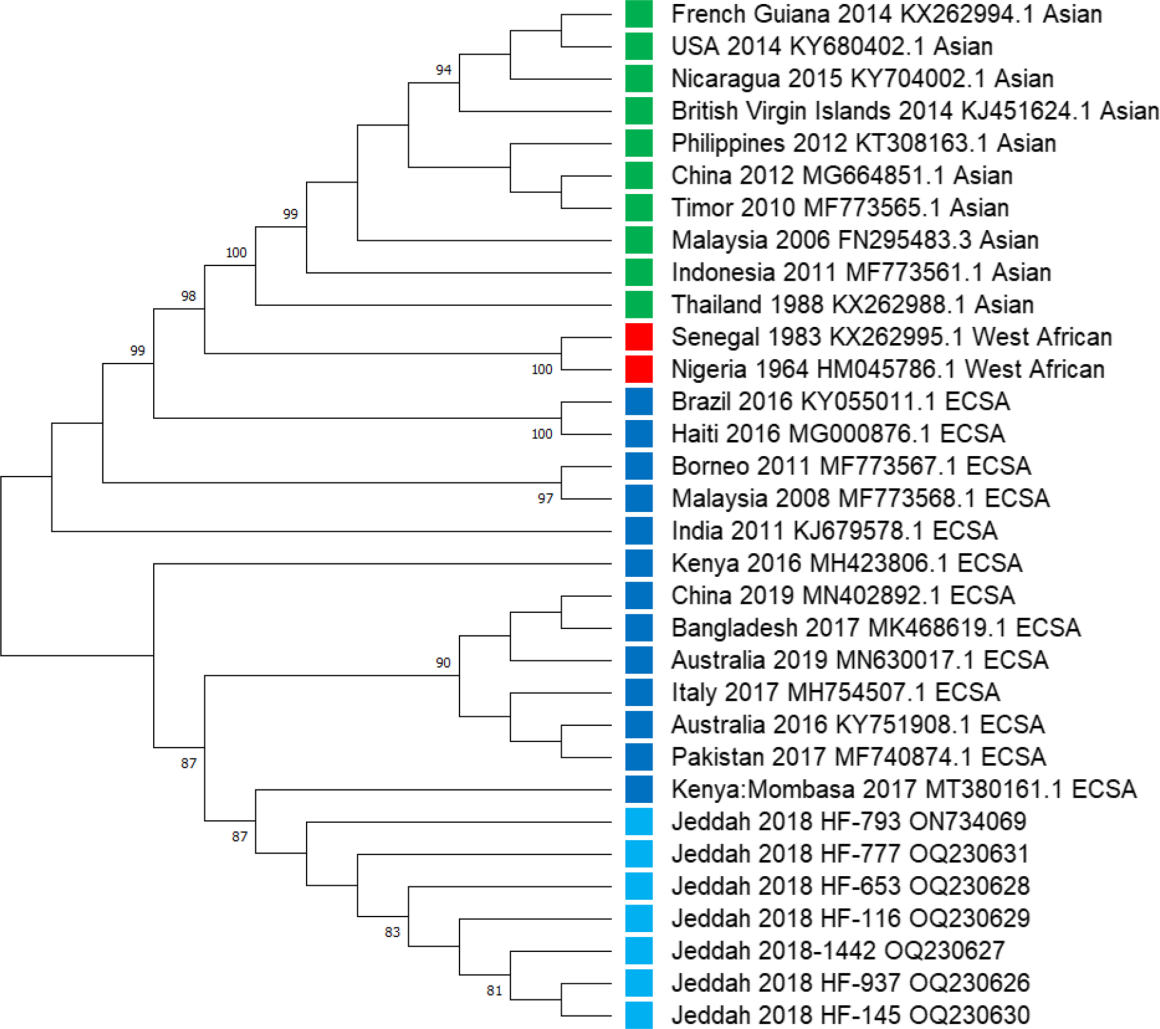
Phylogenetic tree of CHIKV E1 gene from Jeddah during 2018 (light blue squares) mapped to ECSA genotype. Blue circles represent the ECSA genotype. Red circles represent West African genotype; green circles represent Asian genotype. Percent bootstrap support is indicated by the values at each node (the values < 80 are omitted)

**Fig. 3:**
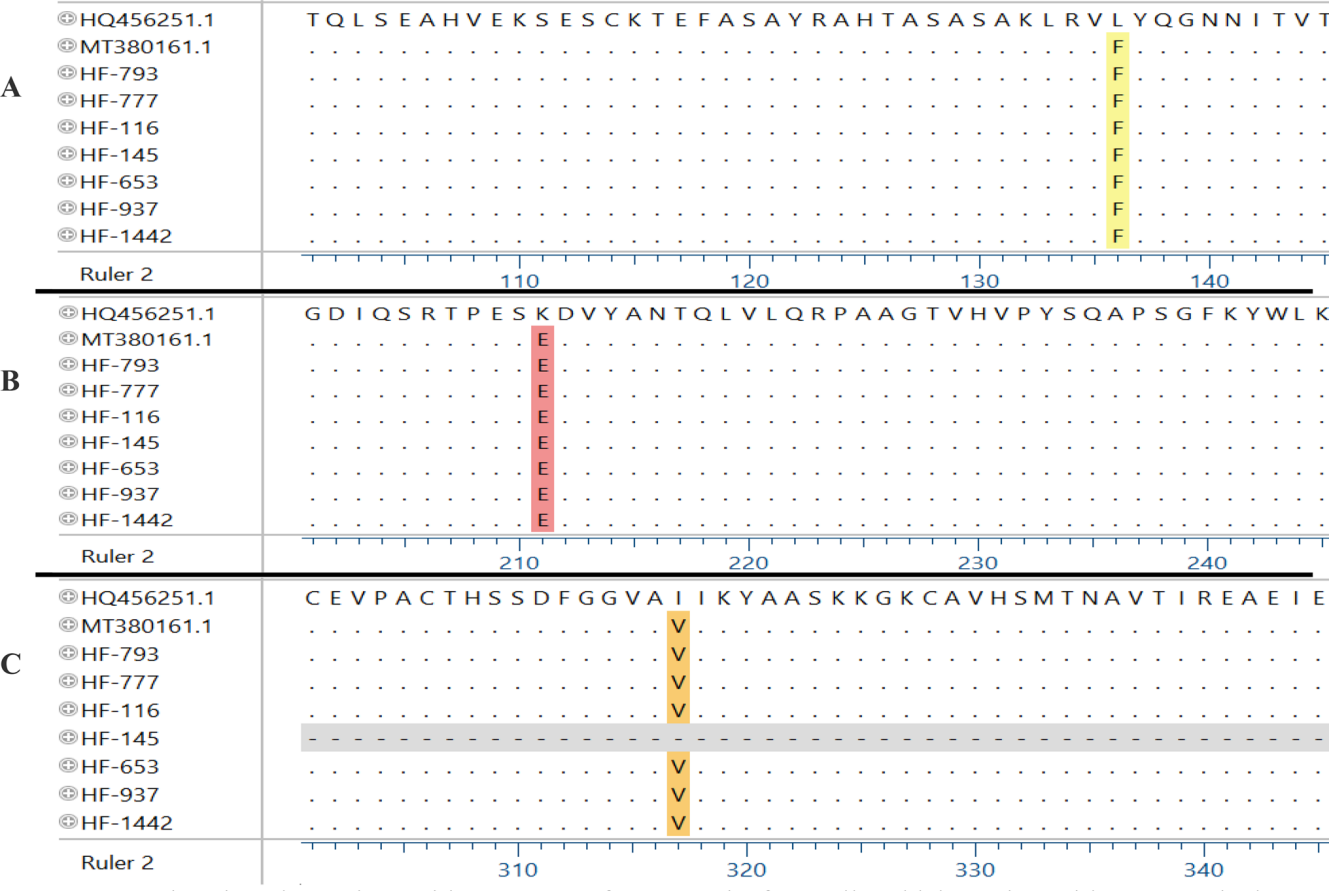
Estimating the amino acid sequence of E1 protein from all Jeddah strains with Kenyan isolate (MT380161.1) and HQ456251.1 as a reference of the ECSA genotype. **A** L136F and **B** K211E were identified in all Jeddah strains. **C** I317V substitutions were identified in all Jeddah strains except sample HF-145 as it produced a short amino acid sequence. The figure was generated with DNASTAR software

**Fig. 4:**
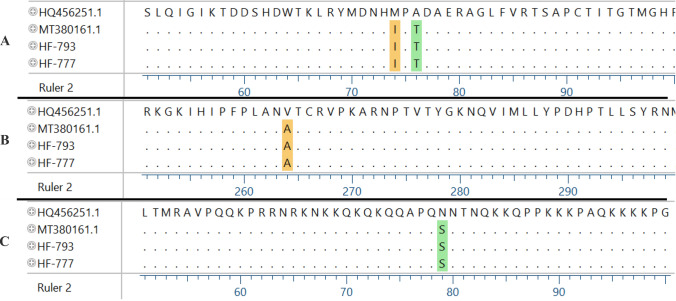
Estimating an amino acid sequence of E2 and C protein from two Jeddah strains (HF-793 and HF-777) with Kenyan isolate (MT380161.1) and HQ456251.1 as a reference of ECSA genotype. **A** E2: M74I and A76T were found in addition to **B** E2: V264A were detected. **C** C: N79S substitutions were identified. The figure was generated with DNASTAR software

As shown in Fig. 1, the construction of the phylogenetic tree of CHIKV Jeddah strains (HF-777 and HF-793) and the 34 other references based on near-full length genome showed that the CHIKV Jeddah 2018 strains (specified by a light blue square) clustered with the CHIKV strains isolated from Kenya mostly during 2017 (such as CHIKV with Gene Bank number MT380161.1). Phylogenetic analysis of the E1 gene of Jeddah strains showed that all CHIKV strains in this study mapped to ECSA genotype (Fig. 2). In the current study, comparing the amino acid of the E1 gene from Jeddah strains with other strains, including the Kenyan isolate (MT380161.1) and HQ456251.1 as a reference for ECSA, revealed that L136F, K211E and I317V substitutions were identified (Fig. 3). Also, M269V and D284E mutations were seen. A226V mutation was not detected in Jeddah strains. Moreover, an assessment of amino acid substitutions of E2 from two samples (HF-793 and HF-777) showed M74I, A76T and V264A mutations (Fig. 4A and B). A study of the E3 amino acids did not show significant substitution. Finally, this study's analysis of amino acid substitutions of C exhibited N79S substitution (Fig. 4C).

## Discussion

CHIKV is an arthropod-borne virus with epidemic potential affecting millions worldwide [14]. Numerous CHIKV re-emergences have been documented in Africa and Asia, with irregular intervals of 2–20 years between outbreaks [15]. CHIKV is endemic to Africa, India and Southeast Asia. Transmission occurs through a bite by an infected *Aedes aegypti* or *Aedes albopictus* and, in some cases, results from maternal–fetal transmission. The spread of CHIKV across countries and continents is attributed to infected travelers from endemic areas [1]. The first known case of autochthonous acute CHIKV in Saudi Arabia (KSA) was reported in 2011 in Jeddah. In this case report the patient presented generalized arthralgia, fever, abdominal pain accompanied by vomiting and skin rash. The analysis of the CHIKV E1 gene showed that the strain belongs to the wild type (Ala 226) [10]. In the current study, we performed the first near-full length genome sequencing on two samples and partial sequencing of the E1 gene on five samples collected from patients in Jeddah in 2018. Phylogenetic analysis showed that CHIKV strains of Jeddah 2018 clustered with strains isolated in 2017 from Kenya, where there was an outbreak in Mombasa (Kenya) between 2017 and 2018 [16]. Out of the 7 patients whose samples were fully or partially sequenced, we obtained the medical records of only four. In summary, their records demonstrated cartilage degeneration, malaise, fever, arthralgia, and acute abdominal pain. These findings are consistent with what were reported worldwide [17]. Dengue infection, also transmitted by the *Aedes* mosquito, remains a significant public health concern in the Western (Jeddah and Makkah) and Southwestern areas (Jazan) of KSA. The *Aedes* mosquito species populations in the western region of Saudi Arabia are abundant, therefore increasing the susceptibility of humans to several arboviruses, including the Chikungunya virus [18]. In addition, the western region in KSA is a destination of millions of travelers (coming from over 180 countries across the globe), including Hajj and Omrah pilgrims, expatriate workers and tourists. These factors significantly increase the risk of the Chikungunya virus establishing endemicity in KSA, which, therefore, launched a vector-control program that had considerable success in the past in controlling *A. aegypti* and, consequently, Dengue fever and dengue hemorrhagic fever. However, the sustained suppression of vector populations largely depends on commitment and community participation in control measures.

The severity and frequency of CHIKV outbreaks highlight the demand for characterization and understanding of the biology of CHIKV [9]. Fitness and transmission of CHIKV in different vectors and hosts have been recognized to be linked with the adaptive mutations that occurred in certain evolutionary lineages [6]. Based on phylogenetic studies CHIKV can be classified into lineages, which are Asian, ECSA and West African [6]. CHIKV strains in the current study showed high similarity and mapped to ECSA genotype. The Indian Ocean Lineage (IOL) of ECSA is characterized by carrying E1: A226V mutation. However, it has been documented that IOL lacked E1:A226V during its recent emergence between 2016 and 2020. Nevertheless, this lineage possesses E1:K211E and E2: V264A substitutions, which led to sporadic emergences of the virus during 2010–2015 in India and 2016 in Kenya. Additionally, acquiring E1: I317V substitution caused massive expansion of this new sub-lineage worldwide in 2014–2020. IOL sub-lineage characterized based on the geographical circulation of the virus by carrying E1:K211E and E2: V264A in addition to E1311V diverged into two main phylogenetic clades, which are Indian subcontinent/Southeast Asia (IS) and Indian subcontinent/Eastern African (IE). IE clade included viruses from Kenya 2014–2018, Sudan 2018, India 2016–2019, Maldives 2019 and Djibouti 2019. IE clade viruses carried E2: A76T and C: N79S. Furthermore, IE involved two different subclades that are Eastern Africa (IE-a) subclade and the Maldives (IE-b) subclade. IE-a carried E2: M74I mutation that characterize the Kenyan (2014–2018), Sudanese (2018) and Djibouti (2019) strains [19]. In the present study, E1 A226V mutation, which is linked with adaptation and enhancement of infectivity of the virus by *Aedes albopictus* [20], was not detected in our strains. Remarkably, in the present study, three amino acid substitutions in the E1 gene were identified that are L136F (Mombasa unique substitution), K211E and I317V, likewise, E2: M74I, A76T and V264A as well as C: A79T were also recognized, that were detected in Kenyan (Mombasa 2017- 2018) isolates. Interestingly these mutations (E1: K211E, and E2: V264A) enhance the CHIKV fitness to the *Aedes aegypti* mosquito [16]. Significantly, it was demonstrated that viruses with these double mutations (E1: K211E, and E2: V264A) in comparison to E1: A226 viruses, have a higher fitness for *Aedes aegypti* through increasing infectivity, dissemination, and transmission by 13, 15 and 62 fold respectively [21]. Based on the current study’s results, CHIKV strains detected in 2018 in Jeddah involved IE—a subclade.

The E1 V80A mutation has been identified for the first time in Kenyan strains and may have the advantage of enhancing transmissibility by *Aedes albopictus* through modification of the demand for cholesterol use [16]. Fortunately, this mutation (V80A) was not detected in the Jeddah strains, as Jeddah city is one of the regions in Saudi Arabia where the *Aedes albopictus* is commonly found [10]. The outcomes of our study may provide useful knowledge about the origin and genetic characteristics of CHIKV. Significantly, investigation of mutations that cause epidemics and novel mutations may help forecast future outbreaks of CHIKV [22]. Therefore, future studies should be continually conducted.

## Conclusion

This study concluded that the CHIKV strains that were detected in Jeddah in 2018 belong to the ECSA genotype. Similar to what has been reported in Kenya during the Mombasa outbreak 2017–2018 [16], E1: L136F, K211E and I317V, E2: M74I, A76T and V264A and C: A79T mutations were identified. Based on these genetic features CHIKV in the present study was IE-a subclade. Consequently, the virus might re-emerge in KSA following the Kenyan (Mombasa) outbreak. Limitations of this study included that there were a limited number of samples, and that the study was conducted retrospectively.

## Supplementary Information

Below is the link to the electronic supplementary material.

Supplementary file1 (DOCX 845 KB)

## Data Availability

Raw data are available upon reasonable request from corresponding author.

## Abbreviation

CHIKV: Chikungunya virus
ECSA: East/Central/South African
RT-PCR: Reverse transcription-polymerase chain reaction
ELISA: Enzyme-linked immunoassay
CT: Cycle threshold
Nsp: Non-structural proteins
bp: Base Pair
IOL: The Indian Ocean Lineage
IS: Indian subcontinent/Southeast Asia
IE: Indian subcontinent/Eastern African
IE-a: Eastern Africa Subclade
IE-b: Maldives Subclade
RRV: Ross river virus
SFV: Semliki forest virus
SINV: Sindbis virus
