# Supplementary material for: The First Genomic Characterization of the Chikungunya Virus in Saudi Arabia
Source: J Epidemiol Glob Health. 2023 Apr 8;13(2):191–9. doi: 10.1007/s44197-023-00098-0 (PMC10272072; doi:10.1007/s44197-023-00098-0)
Supplement: Supplementary file 1 — Supplementary file1 (DOCX 845 KB) [file 44197_2023_98_MOESM1_ESM.docx]

The first genomic characterization of the Chikungunya virus in Saudi Arabia

**Table S1:** Primers sequences of near-full length genome

| **SET** | **FORWARD PRIMER: LENGTH** | | **REVERESE PRIMER: LENGTH** | |
| --- | --- | --- | --- | --- |
| CHIK 1 | TGCGTACCCCATGTTTGAGG | 20 | TTGCCTCGTCTACCTTCTGTC | 21 |
| CHIK 2 | CTGGGTAGGGTTCGACACAA | 20 | TCAGGCCTCTTGTAGACCGT | 20 |
| CHIK 3 | GCCTTCAGTAAGTGGGCGAA | 20 | CATCGTTGCGCTTTCGCTTA | 20 |
| CHIK 4 | TAGCGGAGCAAGTGAAGACG | 20 | GGCGATCAAGGCAAGTAACG | 20 |
| CHIK 5 | TGCCAAGAAATCAGCACCGA | 20 | GCAGATGCCCGCCATTATCG | 20 |
| CHIK 6 | TCTCTGGTGACCCGTGGATA | 20 | CTTTTACTGGGCGGTGTTCG | 20 |
| CHIK 7 | TGTTCGGATTCAACCCTGAG | 20 | ACCACGCACTCTTCATCGTT | 20 |
| CHIK 8 | GGCTCTCTATTGATCAGAGC | 20 | GTCTATGGAGATGTGCTCA | 19 |
| CHIK 9 | CTAACCCAGTCACTGAACCA | 20 | GTATTCCCTTGGACTTACGC | 20 |
| CHIK 10 | TCCCGTGCCTTTGCCGTTAT | 20 | ATGTGATGGGGAACGTCTCG | 20 |
| CHIK 11 | TGTCCGACCGTACAAGAAAC | 20 | ATCGGAGGCGAGTACACAGG | 20 |
| CHIK 12 | GTCCACGGCCAATAGAAGCA | 20 | TAGGTACGCTGTTGCCAAGG | 20 |
| CHIK 13 | ATTTGCTGCCAGCCCTATCA | 20 | GGATTTCATCATAGCGCCGA | 20 |
| CHIK 14 | AGAGGCTGCTTTTGGAGAGA | 20 | CGAGGCTGGTACCTCCTATTG | 21 |
| CHIK 15 | CAGGTGACGAACAAGACGAAG | 21 | CTTAGATGACCGCTTGAAGGC | 21 |
| CHIK 16 | AGTCAAGCATGAAGGTAAGGT | 21 | GGATTTTCAACGTCCCGTCTG | 21 |
| CHIK 17 | ACACCCTGCTGCTACGAAAA | 20 | CCGCACCGTCTGACTATTGA | 20 |
| CHIK 18 | AGGGAACTACCTTGCAGCAC | 20 | TGTGGATAACTGCGGCCAAT | 20 |
| CHIK 19 | AAGAAGAGTGGGTGACGCAT | 20 | TACGGAGACGGGATAACGGT | 20 |
| CHIK 20 | CGCGTACGAACACGTAACAG | 20 | GATGGTGCCTGAGAGTACGG | 20 |
| CHIK 21 | CCGTCACAGTTAAGGACGCT | 20 | CCACACCTCCCGTGATCTTC | 20 |
| CHIK 22 | GAGTGCCATCCACCGAAAGA | 20 | AGCCCTTTGAACTACTTCT | 19 |

**Table S2:** Primers sequences of second round (nested) PCR

| **SET** | **FORWARD PRIMER: LENGTH** | | **REVERESE PRIMER: LENGTH** | |
| --- | --- | --- | --- | --- |
| CHIK E1 | GCCTACTGCTTCTGCGACA | 19 | CGCTCTTACCGGGTTTGTTG | 20 |

**Table S3:** The result of CHIKV PCR of the provided samples

| **Sample** | **CHIKV real-time PCR** | **Ct** | **Detection band of interest** | **Detection of 2^nd^ round** |
| --- | --- | --- | --- | --- |
| **Samples used for whole-genome sequencing** | | | | |
| **HF-777** | CHIKV RNA Detected | 26 | **All 22 bands detected*** | Not done |
| **HF-793** | CHIKV RNA Detected | 17 | **All 22 bands detected*** | Not done |
| **Samples used for (partial) E1 gene sequencing** | | | | |
| **HF-116** | CHIKV RNA Detected | 26 | **Detected (1267 bp)**** | Not done |
| **HF-145** | CHIKV RNA Detected | 31 | Detected (1267 bp) faint band | **Detected 530 bp***** |
| **HF-653** | CHIKV RNA Detected | 18 | **Detected (1267 bp)**** | Not done |
| **HF-730** | CHIKV RNA Detected | 32 | Not detected | Not detected |
| **HF-748** | CHIKV RNA Detected | 33 | Detected (1267 bp) faint band | Detected 530 bp Faint band |
| **HF-754** | CHIKV RNA Detected | 36 | Not detected | Not detected |
| **HF-755** | CHIKV RNA Detected | 32 | Detected (1267 bp) faint band | Detected 530 bp Faint band |
| **HF-760** | CHIKV RNA Detected | 33 | Not detected | Not detected |
| **HF-764** | CHIKV RNA Detected | 33 | Detected (1267 bp) faint band | Detected 530 bp Faint band |
| **HF-770** | CHIKV RNA Detected | 33 | Not detected | Detected 530 bp Faint band |
| **HF-772** | CHIKV RNA Detected | 33 | Not detected | Detected 530 bp Faint band |
| **HF-773** | CHIKV RNA Detected | 33 | Not detected | Not detected |
| **HF-775** | CHIKV RNA Detected | 34 | Not detected | Not detected |
| **HF-776** | CHIKV RNA Detected | 33 | Not detected | Detected 530 bp Faint band |
| **HF-778** | CHIKV RNA Detected | 33 | Not detected | Detected 530 bp Faint band |
| **HF-937** | CHIKV RNA Detected | 18 | **Detected (1267 bp)**** | Not done |
| **HF-1018** | CHIKV RNA Detected | 34 | Not detected | Not detected |
| **HF-1083** | CHIKV RNA Detected | 32 | Not detected | Not detected |
| **HF-1442** | CHIKV RNA Detected | 21 | **Detected (1267 bp)**** | Not done |

*Represent samples in large volume and used for studying whole genome sequence. **Represents samples that were used for studying (partial) the E1 sequence. ***Represents the sample that was used for studying (partial) the E1 sequence from nested PCR. The remaining samples were not suitable for the sequencing step due to low viral low.

**Table S4:** Result of all CHIKV strains genome from Jeddah in 2018

| **Sample** | **Length (bp)** | **The accession number of the most similar strain and source** | **Identity (%)** |
| --- | --- | --- | --- |
| **Samples used for whole genome sequencing** | | | |
| HF-793 | 11492 | MT380161.1 from "Kenya: Mombasa 2017 | 99.90 |
| HF-777 | 11097 | MT380161.1 from "Kenya: Mombasa 2017 | 99.90 |
| **Samples used for (partial) E1 gene sequencing** | | | |
| HF-116 | 1161 | MT380161.1 from "Kenya: Mombasa 2017 | 99.91 |
| HF-653 | 1162 | MT380161.1 from "Kenya: Mombasa 2017 | 100 |
| HF-937 | 1159 | MT380161.1 from "Kenya: Mombasa 2017 | 99.83 |
| HF-1442 | 1159 | MT380161.1 from "Kenya: Mombasa 2017 | 100 |
| HF-145 | 519 | MT380161.1 from "Kenya: Mombasa 2017 | 99.42 |


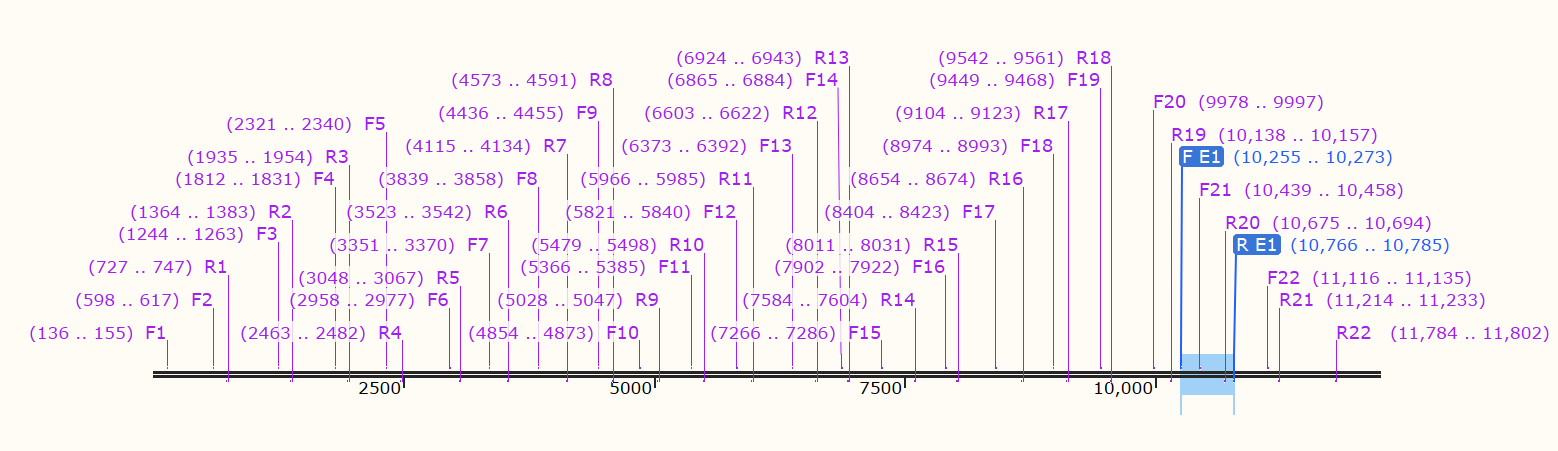


**Figure S1:** A map of primers positions**.** The highlighted area represents the part of CHIKV genome, which was amplified by nested PCR.


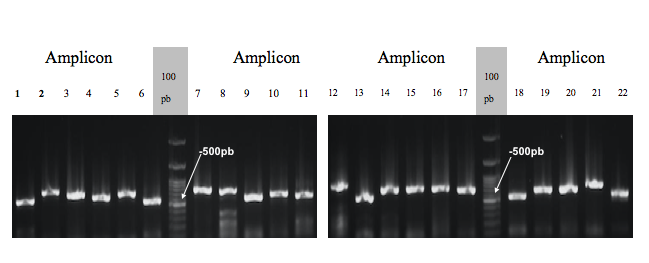


**Figure S2:** Gel electrophoresis of PCR products representing CHIKV genome from Jeddah 2018. Bands from 1 to 22 represent PCR products from the same sample.


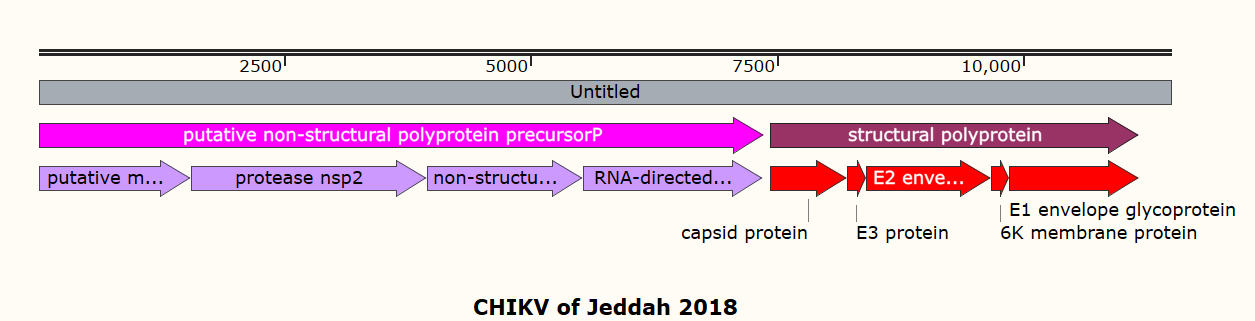


**Figure S3:** The annotation of the CHIKV full sequence of Jeddah strain in 2018 via BV-BRC revealed a total of 9 genes: E1, 6K, E2, E3, capsid, RNA-directed RNA polymerase nsp4, non-structural protein nsp3, protease nsp2 and putative mRNA-capping enzyme nsp1, C-terminal. The figure was generated with SnapGene viewer.
